# Sperm-carried RNAs play critical roles in mouse embryonic development

**DOI:** 10.18632/oncotarget.18672

**Published:** 2017-06-27

**Authors:** Lei Guo, Shi-Bin Chao, Lu Xiao, Zhen-Bo Wang, Tie-Gang Meng, Yuan-Yuan Li, Zhi-Ming Han, Ying-Chun Ouyang, Yi Hou, Qing-Yuan Sun, Xiang-Hong Ou

**Affiliations:** ^1^ Center for Reproductive Medicine, Guangdong Second Provincial General Hospital, Guangzhou 510317, PR China; ^2^ State Key Laboratory of Stem Cell and Reproductive Biology, Institute of Zoology, Chinese Academy of Sciences, Beijing 100101, PR China; ^3^ The ART Center, Jiujiang Maternal and Child Health Care Hospital, Jiangxi 332000, PR China; ^4^ Department of Obstetrics and Gynecology, Nanfang Hospital, Southern Medical University, Guangzhou 510515, PR China; ^5^ College of Life Sciences, University of Chinese Academy of Sciences, Beijing 100049, PR China

**Keywords:** sperm RNA, RNase, intracytoplasmic sperm injection, blastocyst formation, offspring

## Abstract

Recently, numerous studies have reported that the mature sperm contains both coding and non-coding RNAs and the sperm delivers some RNAs to the oocyte at fertilization. However, the functions of the RNAs carried to the oocyte by sperm at fertilization in embryonic development remains a mystery. In this study, the mature spermatozoa were treated with lysolecithin, pronase and RNases (RNase A and RNase H) to remove the sperm-carried RNAs, and then injected into normal mature oocyte. The results showed that after the treatment, the content of the sperm RNAs was decreased by about 90%. The blastocyst formation rate and the live birth rate of the embryos from intracytoplasmic sperm injection (ICSI) using the treated sperm were significantly decreased (*P*<0.01), while these effects were partially rescued by injecting total wide-type sperm RNAs. The reproductive capacity of offspring (F0) in sperm-treated group was similar with that in control group (*P*>0.05), but the body weight of F1 mice from sperm-treated group was lower than that in control group after two weeks of birth (*P*<0.05). These results demonstrated that the sperm-carried RNAs have important roles in embryonic development.

## INTRODUCTION

Meiosis is a series of nuclear and cytoplasmic events that produce a haploid gamete which has the capability to complete fertilization and then establish an embryo. The maturing oocyte undergoes a series of dynamic morphological and nuclear rearrangements, during which its transcriptional activity sharply decreases, but oocyte accumulates maternal RNAs that will ensure early stages of embryogenesis until zygotic genome activation [1]. The production of mature male gametes is also a complex process. Spermatogonia differentiate to primary spermatocytes, then though meiosis, the round spermatids are formed [2]. At the final step of spermiogenesis, the majority of the cytoplasm with most RNAs is depleted; meanwhile the nuclear histones are replaced by protamines. Finally, the sperm becomes a highly differentiated transcriptionally inactive specialized cell containing minimal cytoplasm and compacted nucleus [3].

In classical concept, almost all the necessary cytoplasmic components for early embryonic development are from oocyte, and the only contribution of sperm is paternal genomic DNA. Recently, numerous studies have reported the existence of sperm RNA in mammals, including rat [4], mouse [5], and human [6, 7]. Ostermeier et al. found that a human sperm RNA profile contains about 3000–7000 types of coding transcripts using microarrays [8]. Next-generation sequencing technique provides a more complete picture of transcript population in mature spermatozoa, including both known and unknown transcripts [9, 10]. RNA-seq analysis showed that the mature sperm contains both coding and non-coding RNAs that include both fragmented and non-degraded mRNAs, siRNAs, miRNAs, piRNAs and long non-coding RNAs [11].

There is no doubt that the sperm delivers some RNAs to the oocyte at fertilization. The individual functions of some transcripts in mature sperm have been elucidated [12, 13], but the functions of the RNAs carried to the oocyte by sperm at fertilization in embryonic development remains a mystery. In this study, the mature spermatozoa were treated with lysolecithin (LL), pronase (P) and RNases (RNase A and RNase H) to remove the sperm-carried RNAs, and then injected into normal mature oocytes to observe the functions of sperm RNAs in embryo development. The blastocyst formation and the offspring were detected.

## RESULTS

### Sperm RNAs after LL+P+RNases treatment

**Figure 1:**
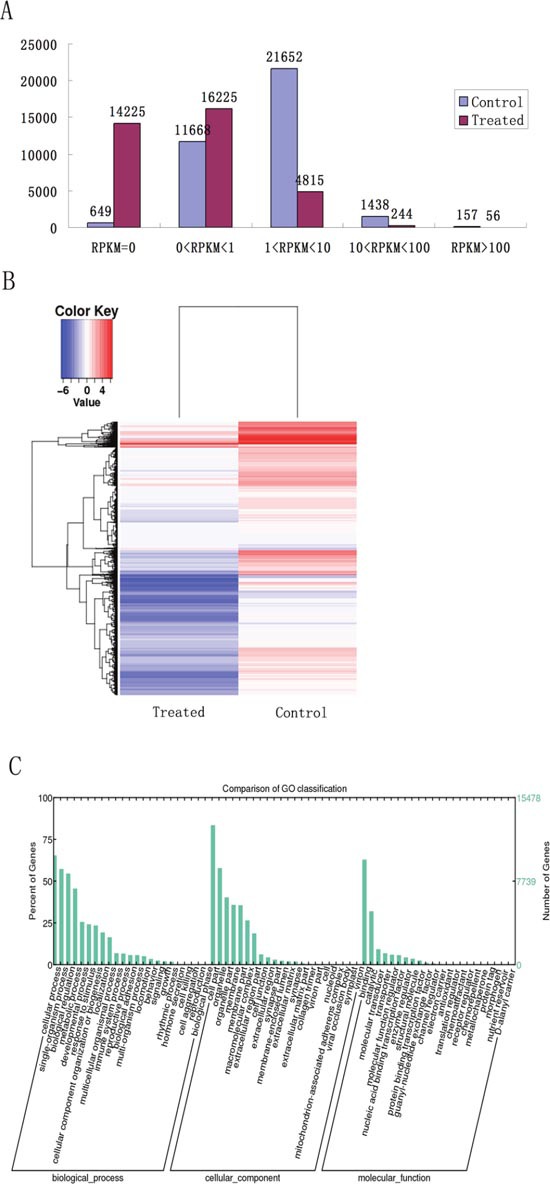
Analysis of RNA-seq data from the sperm with or without LL+P+RNases-treatment **(A)** Distribution of RPKM from the sperm with or without treatment. **(B)** Heat maps derived from cluster analysis of RNA-seq from the sperm with or without treatment. **(C)** The gene ontology analysis of the differentially expressed RNAs from the sperm with or without treatment.

To remove RNAs, the spermatozoa were treated with lysolecithin, pronase and RNases (RNase A and RNase H). After the treatment, the content of sperm RNAs was decreased by about 90% (53.64±10.67 ng/10^7^ VS 538.88±34.16 ng/10^7^). To detect whether all RNAs were reduced, RNA-seq was performed. A total of 35565 transcripts were obtained from RNA-seq. Compared to control group, the contents of 20083 transcripts were lower, and 14225 transcripts were not detected in treated group (Figure 1A and Figure 1B). The gene ontology analysis found that 44.95%, 16.41% and 38.64% of the transcripts encoding proteins were related to the biological process, molecular function and cellular component, respectively (Figure 1C). In the biological process, 3893 transcripts were involved in development process and 922 were related to reproductive process, including 6 mRNAs encoding proteins associated with embryonic cleavage (GO: 0040016), 30 with blastocyst formation (GO:0001825), 61 with blastocyst development (GO:0001824) and 21 with blastocyst growth (GO:0001832) (Table 1).

**Table 1:** The ontological categories of different expressive transcripts associated with embryonic cleavage, blastocyst formation, blastocyst development and blastocyst growth

GO term	Description	No. of genes	Genes
GO:0040016	embryonic cleavage	6	Cullin-3, Protein AATF, DNA topoisomerase 2-alpha,Phosphatidylinositol 4,5-bisphosphate 3-kinase catalytic subunit beta isoform, DNA topoisomerase 1, TFIIH basal transcription factor complex helicase XPD subunit
GO:0001825	blastocyst formation	30	14 kDa phosphohistidine phosphatase, Adenosine deaminase, B-cell lymphoma/leukemia 10, Butyrophilin subfamily 2 member A2, Butyrophilin-like protein 2, C-C chemokine receptor type 7, CKLF-like MARVEL transmembrane domain-containing protein 3, CMRF35-like molecule 8, Dual specificity protein phosphatase 22, E3 ubiquitin-protein ligase CBL-B, ETS-related transcription factor Elf-1, Fc receptor-like protein 5, Germinal center-associated signaling and motility protein, Intermediate conductance calcium-activated potassium channel protein 4, Leupaxin, Mucosa-associated lymphoid tissue lymphoma translocation protein 1 homolog, NFAT activation molecule 1, Protein TESPA1, Receptor-type tyrosine-protein phosphatase C, Roquin-1, Serine/threonine-protein kinase D2, Signal-transducing adaptor protein 1, Thy-1 membrane glycoprotein, Transcription factor Sp1, Tyrosine-protein kinase Lyn, Tyrosine-protein phosphatase non-receptor type 2, Tyrosine-protein phosphatase non-receptor type 6, Ubiquitin-associated and SH3 domain-containing protein A, Voltage-dependent L-type calcium channel subunit alpha-1F, Zona pellucida sperm-binding protein 3
GO:0001824	blastocyst development	61	60S ribosomal protein L7-like 1, Activin receptor type-1C, Adenosine deaminase, BCL2/adenovirus E1B 19 kDa protein-interacting protein 2, Breast cancer type 2 susceptibility protein homolog, Bromodomain-containing protein 4, Bystin, Cbp/p300-interacting transactivator 2, CCR4-NOT transcription complex subunit 1, CCR4-NOT transcription complex subunit 3, Condensin-2 complex subunit G2, Deleted in malignant brain tumors 1 protein, DNA endonuclease RBBP8, DNA repair protein RAD51 homolog 2, DNA replication complex GINS protein PSF1, DNA replication complex GINS protein SLD5, Eomesodermin homolog, ETS translocation variant 2, Forkhead box protein D3, G2/M phase-specific E3 ubiquitin-protein ligase, Heart- and neural crest derivatives-expressed protein 1, Hepatocyte nuclear factor 1-alpha, Histone-lysine N-methyltransferase SETDB1, Homeobox protein CDX-2, Homeodomain-only protein, HORMA domain-containing protein 1, Integrator complex subunit 1, Lysine-specific demethylase 4C, Mediator of RNA polymerase II transcription subunit 21, Methylcytosine dioxygenase TET1, NEDD4-binding protein 2-like 2, Nodal, Notchless protein homolog 1, Nuclear autoantigenic sperm protein, Nuclear distribution protein nudE-like 1, Partner and localizer of BRCA2, POU domain class 5 transcription factor 1, Pre-mRNA-processing factor 19, Pre-mRNA-splicing factor SYF1, Proheparin-binding EGF-like growth factor, Ribosomal RNA small subunit methyltransferase NEP1, RNA polymerase-associated protein RTF1 homolog, Sal-like protein 4, Serine/threonine-protein kinase Nek2, Ski-like protein, Splicing factor 3B subunit 6, Steroid hormone receptor ERR2, SWI/SNF-related matrix-associated actin-dependent regulator of chromatin subfamily B member 1, THO complex subunit 2, THO complex subunit 5 homolog, Transcription factor AP-2 gamma, Transcription factor SOX-17, Transcription factor Sp1, Transcription factor Sp3, Transcription factor Spi-C, Transforming growth factor beta receptor type 3, Upstream-binding factor 1-like protein 1, WD repeat-containing protein 74, Zinc finger protein 830, Zinc finger protein ZPR1, Zona pellucida sperm-binding protein 3
GO:0001832	blastocyst growth	21	Activin receptor type-1C, Breast cancer type 2 susceptibility protein homolog, Bromodomain-containing protein 4, Condensin-2 complex subunit G2, Deleted in malignant brain tumors 1 protein, DNA repair protein RAD51 homolog 2, DNA replication complex GINS protein PSF1, DNA replication complex GINS protein SLD5, Histone-lysine N-methyltransferase SETDB1, Integrator complex subunit 1, Nuclear distribution protein nudE-like 1, Partner and localizer of BRCA2, POU domain class 5 transcription factor 1, Pre-mRNA-processing factor 19, Proheparin-binding EGF-like growth factor, RNA polymerase-associated protein RTF1 homolog, Sal-like protein 4, Steroid hormone receptor ERR2, Upstream-binding factor 1-like protein 1, Zinc finger protein 830, Zinc finger protein ZPR1

### Oocytes fertilized by ICSI using spermatozoa treated with LL+P+RNases display reduced developmental potential

To test whether sperm with low RNA contents could support fertilization and early embryonic development, we respectively injected the control sperm and the sperm treated with LL+P+RNases into oocytes (C57BL/6J), and counted the number of embryos at each preimplantation developmental stage to evaluate the developmental potential. The blastocyst formation rate of embryos from treated sperm was significantly reduced, compared to the control group (*P*<0.01) (Table 2). To evaluate the post-implantation development of the embryos derived from treated sperm, two-cell embryos were transferred into the oviducts of recipient mice to observe the development to term. The live birth rate of embryos in the control group was significantly higher than that in the treated group (36.67% *VS* 3.21%, *P*<0.01) (Table 3), suggesting that the developmental potential of embryos derived from ICSI using RNA-deficient spermatozoa was decraesed.

**Table 2:** Preimplantation development of embryos derived from ICSI using WT oocytes and LL+P+RNases-treated sperm with or without total WT sperm RNA

	No. of oocytes injected	Number of embryos at each stage				
		2PN(% of total)	2-cell(% of 2PN)	4-8-cell(% of 2-cell)	Morula(% of 2-cell)	Blastocyst(% of 2-cell)
Control	156	151 (96.79)	125 (82.78)	116 (92.80)	102 (81.60)	70 (56.00)
LL+P+RNases-treated	227	218 (96.04)	177 (81.19)	161 (90.96)	139 (78.53)	28 (15.82)^**, ##^
LL+P+RNases-treated + WT sperm RNA	82	77 (93.9)	67 (87.01)	61 (91.04)	56 (83.58)	27 (40.30)

^**^ LL+P+RNases-treated group VS control group, *P* < 0.01.

^##^ LL+P+RNases-treated + WT sperm RNA group VS LL+P+RNase-treated group, *P* < 0.01.

**Table 3:** Term development of mouse embryos developed from the oocytes fertilized by injection of LL+P+RNases-treated sperm with or without total WT sperm RNA

	No. of 2-cell embryos transferred (No. of recipients)	No. of live offspring at term (%)
Control	60 (4)	22 (36.67)
LL+P+RNases-treated	187 (10)	6 (3.21)**^, ##^
LL+P+RNases-treated + WT sperm RNA	86 (5)	17 (19.77)

^**^ LL+P+RNases-treated group VS control group, *P* < 0.01.

^##^ LL+P+RNases-treated + WT sperm RNA group VS LL+P+RNase-treated group, *P* < 0.01.

### Supplementation of wide-type (WT) sperm RNAs significantly improves the blastocyst formation rate and the birth rate of ICSI embryos derived from the treated sperm

The reduced developmental potential of embryos derived from ICSI using LL+P+RNases-treated sperm might not be due to the deficiency in RNA contents because these sperm underwent a series of treatment *in vitro*. To test whether supplementation of total normal paternal RNAs improves the developmental potential of the embryos, total RNAs isolated from WT sperm were injected into oocyte fertilized by sperm treated with LL+P+RNases through ICSI. After injected total WT sperm RNAs, the blastocyst formation rate of embryos from ICSI with LL+P+RNases-treated sperm was significantly increased than that of the embryos from LL+P+RNases-treated sperm through ICSI without injection of WT sperm RNAs (*P*<0.01) (Table 2). To examine the post-implantation development of the embryos from treated spermatozoa and injected total WT sperm RNAs, 2-cell embryos were transferred into the oviducts of recipient mice. 19.77% of 2-cell transferred embryos from treated spermatozoa and injected with total WT sperm RNAs led to live-born pups, significantly higher than that of the embryos derived from treated spermatozoa without injected total WT sperm RNAs (*P*<0.01) (Table 3). This suggested that supplementation of normal sperm-carried RNAs can partially rescue not only the preimplantation development, but also the birth rate of ICSI embryos from RNA-deficient sperm in mice.

### Expression patterns of H3K27me3, H3K4me3, H3K14ac and H4K12ac in late 1-cell and 2-cell embryos from LL+P+RNases-treated sperm are normal

**Figure 2:**
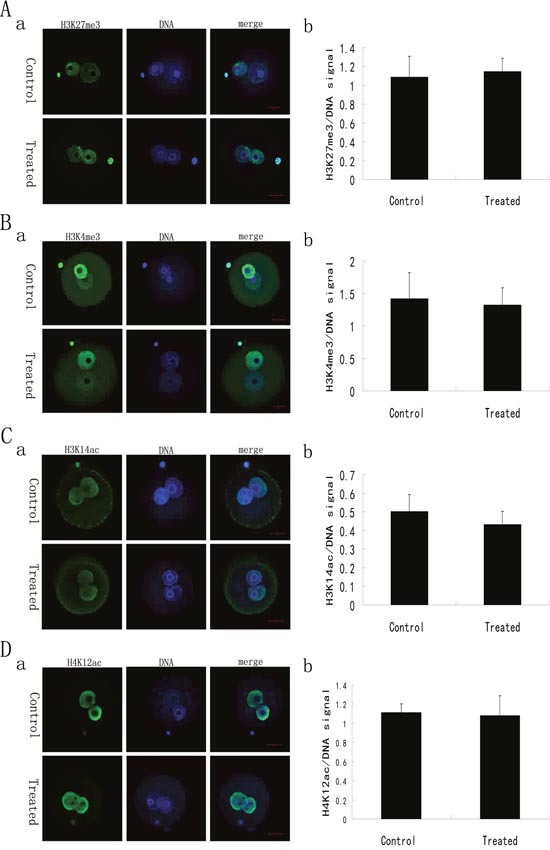
Histone modifications of the late 1-cell stage embryos derived from ICSI using the sperm with or without LL+P+RNases-treatment **(A)** H3K27me3 in the late 1-cell stage embryos derived from ICSI using the sperm with or without treatment (n = 18). The staining pattern of H3K27me3 in the late 1-cell stage embryos derived from ICSI using the sperm with or without treatment **(a)** and the ratio of H3K27me3/DNA signal intensity in the late 1-cell stage embryos derived from ICSI using the sperm with or without treatment **(b)**. H3K27me3: green; DNA: blue. Bar=20μm. **(B)** H3K4me3 in the late 1-cell stage embryos derived from ICSI using the sperm with or without treatment (n = 20). The staining pattern of H3K4me3 in the late 1-cell stage embryos derived from ICSI using the sperm with or without treatment **(a)** and the ratio of H3K4me3/DNA signal intensity in the late 1-cell stage embryos derived from ICSI using the sperm with or without treatment **(b)**. H3K4me3: green; DNA: blue. Bar=20μm. **(C)** H3K14ac in the late 1-cell stage embryos derived from ICSI using the sperm with or without treatment (n = 19). The staining pattern of H3K14ac in the late 1-cell stage embryos derived from ICSI using the sperm with or without treatment **(a)** and the ratio of H3K14ac/DNA signal intensity in the late 1-cell stage embryos derived from ICSI using the sperm with or without treatment **(b)**. H3K14ac: green; DNA: blue. Bar=20μm. **(D)** H4K12ac in the late 1-cell stage embryos derived from ICSI using the sperm with or without treatment (n = 18). The staining pattern of H4K12ac in the late 1-cell stage embryos derived from ICSI using the sperm with or without treatment **(a)** and the ratio of H4K12ac/DNA signal intensity in the late 1-cell stage embryos derived from ICSI using the sperm with or without treatment **(b)**. H4K12ac: green; DNA: blue. Bar=20μm.

**Figure 3:**
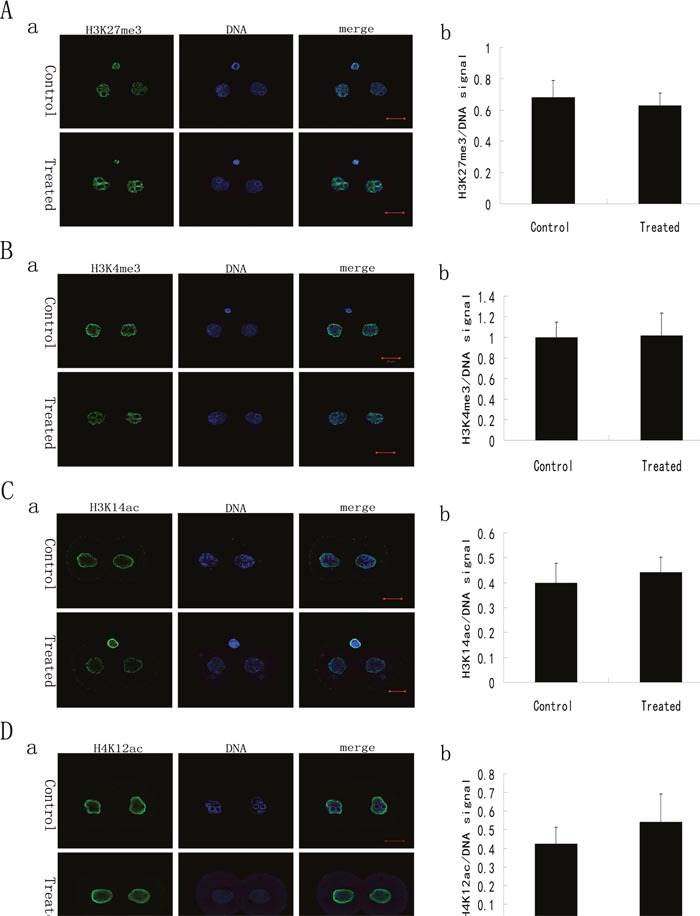
Histone modifications of the 2-cell stage embryos derived from ICSI using the sperm with or without LL+P+RNases-treatment **(A)** H3K27me3 in 2-cell stage embryos derived from ICSI using the sperm with or without treatment (n = 20). The staining pattern of H3K27me3 in 2-cell stage embryos derived from ICSI using the sperm with or without treatment **(a)** and the ratio of H3K27me3/DNA signal intensity in 2-cell stage embryos derived from ICSI using the sperm with or without treatment **(b)**. H3K27me3: green; DNA: blue. Bar=20μm. **(B)** H3K4me3 in 2-cell stage embryos derived from ICSI using the sperm with or without treatment (n = 18). The staining pattern of H3K4me3 in 2-cell stage embryos derived from ICSI using the sperm with or without treatment **(a)** and the ratio of H3K4me3/DNA signal intensity in 2-cell stage embryos derived from ICSI using the sperm with or without treatment **(b)**. H3K4me3: green; DNA: blue. Bar=20μm. **(C)** H3K14ac in 2-cell stage embryos derived from ICSI using the sperm with or without treatment (n = 21). The staining pattern of H3K14ac in 2-cell stage embryos derived from ICSI using the sperm with or without treatment **(a)** and th ratio of H3K14ac/DNA signal intensity in 2-cell stage embryos derived from ICSI using the sperm with or without treatment **(b)**. H3K14ac: green; DNA: blue. Bar=20μm. **(D)** H4K12ac in 2-cell stage embryos derived from ICSI using the sperm with or without treatment (n = 17). The staining pattern of H4K12ac in 2-cell stage embryos derived from ICSI using the sperm with or without treatment **(a)** and the ratio of H4K12ac/DNA signal intensity in 2-cell stage embryos derived from ICSI using the sperm with or without treatment **(b)**. H4K12ac: green; DNA: blue. Bar=20μm.

As an important part of epigenetics, histone modifications play important roles in maintaining higher-order chromatin structure and in regulating various chromatin processes, such as DNA repair, DNA replication and transcription during development [14]. To detect whether histone modifications are affected by an initial lack of sperm-carried RNAs during the zygotic genome activation, the expression patterns of H3K4me3, H3K27me3, H3K14ac and H4K12ac in late 1-cell and 2-cell embryos from ICSI using LL+P+RNases-treated spermatozoa were examined. Immunofluorescence staining showed that H3K4me3, H3K27me3, H3K14ac and H4K12ac were expressed in both male and female pronuclei of late 1-cell embryos and in each nucleus of 2-cell embryos from control group or LL+P+RNases-treated sperm group (Figure 2 and Figure 3). No discernable differences were observed between the two groups.

### Effects of RNA deficiency in sperm on offspring

**Figure 4:**
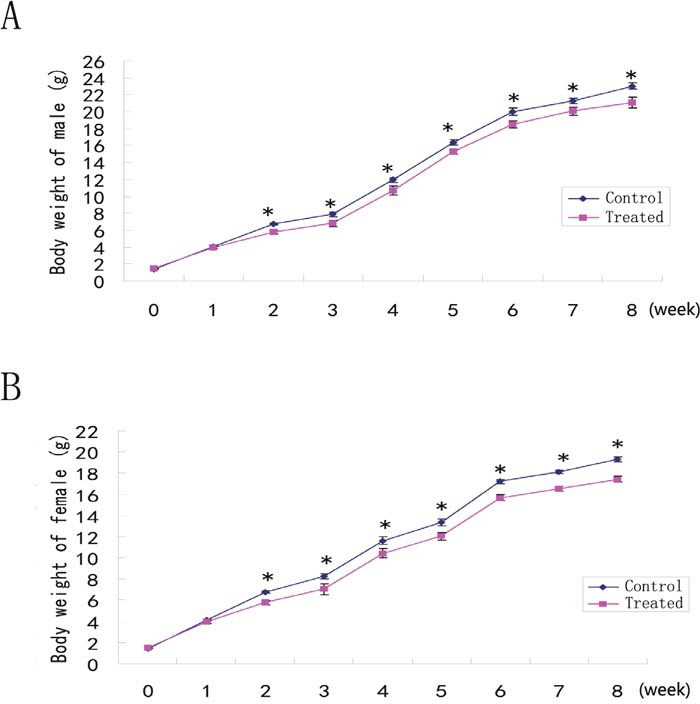
The body weight trajectories of F1 males **(A)** and females **(B)**. * *P*<0.05.

The offspring (F0) from ICSI using LL+P+RNases-treated sperm with or without supplementation of WT sperm RNAs could develop to adulthood normally. To test the effects of RNA-deficient sperm in offspring, the reproductive capacity of offspring (F0) and the body weight trajectories of offspring (F1) were detected. The results showed that the reproductive capacity of F0 females or males in treated group was similar to that in control group (*P*>0.05) (Table 4). However, the body weight of F1 mice from sperm-treated group was lower than that in control group after two weeks of birth (*P*<0.05) (Figure 4).

**Table 4:** The reproductive capacity of F0 mice

		No. of mice examined	No. of total litters	No. of offspring per litter
Female	control	3	19	8.21±0.37
	LL+P+RNases-treated	3	18	7.72±0.38
Male	control	3	20	8.11±0.21
	LL+P+RNases-treated	3	18	7.89±0.43

## DISCUSSION

In this study, we demonstrate that the content of the sperm RNAs was decreased by about 90% after treatment with LL+P+RNases, and that the blastocyst formation rate and the live birth rate were significantly decreased in embryos derived from ICSI using LL+P+RNases-treated sperm, compared to control group. The offspring from ICSI using LL+P+RNases-treated spermatozoa could develop to adulthood normally, and the reproductive capacity of offspring (F0) was similar to that in the control group, but the body weight of F1 mice was lower than that in control group after two weeks of birth.

In the late 1990s, various studies reported the presence of different types of RNAs in mammalian spermatozoa [11]. The spermatozoal RNAs include both fragmented and non-degraded mRNAs, siRNAs, miRNAs, piRNAs and long non-coding RNAs [11]. A much more complete picture of transcript population in mature spermatozoa could be provided using a new next-generation sequencing technique. Approximately 22302 unique transcripts were detected using RNA-seq in human mature sperm [9]. In mouse, RNA-seq showed that the mature sperm had more than 33000 different transcripts, including 27310 coding transcripts [15]. *In situ* hybridization (ISH) studies on spermatozoa have found that RNAs are localized in the entire head region [16], and some ISH data show that the midpiece of sperm tail is another site of RNA deposition [17]. Lysolecithin and pronase were used to remove the acrosomal and plasma membrane and some perinuclear materials [18]. RNase A cleaves nucleoside 3'-phosphates and 3'-phospho-oligonucleotides ending in Cp or Up in single-stranded RNA [19], and the activity of RNase H is the cleavage of RNA in RNA/DNA hybrids [19]. In this study, to remove the sperm RNAs, we treated the mouse mature sperm with lysolecithin and pronase, and then further treated with RNase A and RNase H. The results showed that after treatment, the content of sperm RNAs was reduced by about 90%. Furthermore, RNA-seq analysis showed that 20083 of 35565 transcripts were lower, and 14224 transcripts were not detected in LL+P+RNases-treated sperm, compared with control group. This might because that RNases did not get to the location of some RNAs or the enzyme activity was inhibited to some extent in the sperm nucleus, leading to removal failure of a small fraction of RNAs.

There is no doubt that the sperm delivers some RNAs to the oocyte at fertilization. Three different types of sperm RNAs have been discovered [20]. The first group of RNAs has an important function during spermatogenesis but has no role in post-fertilization events. The presence of this set of RNAs could be used as a diagnostic tool to detect the quality of mature spermatozoa [12, 21, 22]. A second group of RNAs from a non-testicular source can be introduced into the oocyte during fertilization by sperm [23, 24]. The third group of RNAs from the testicular germ cells may have roles in the fertilized egg. Sperm-specific *Plcζ* mRNA injected into the mouse oocyte translated into PLCζ protein and induced functional calcium oscillations [25]. Yao et al reported that sperm-borne *Dby* mRNA regulated the zygotic development after *Dby* mRNA transferred into pronucleus [26]. Meanwhile, some noncoding RNAs in mature spermatozoa might have functions in early embryo development. Injection of miR-34c inhibitor into zygotes inhibits first cleavage division, suggesting that miR-34c has important role in the first cleavage division in mice [27], but the miR-34b/c knockout mice displayed normal fertility [13]. Previous study found that the live birth rate from the oocyte through ICSI using sperm treated with lysolecithin was increased [28], and the rate of live-born normal offspring, using sperm treated with lysolecithin and pronase, was decreased [18]. Pronase treatment may remove some peinuclear materials or some RNAs which play implant roles in embryonic development [18]. In this study, we treated the mature sperm with LL+P+RNases to remove the sperm RNAs, and then injected the treated sperm into oocyte. The blastocyst formation rate and the live birth rate of the embryo from treated sperm were significantly decreased than that in control group (*P*<0.01). When we injected the total WT sperm RNAs into eggs, the blastocyst formation rate and the live birth rate of the embryo from treated sperm were increased, suggesting that total sperm RNAs could rescue the decreased developmental potential of embryo from treated sperm. These results show that deletion of sperm RNAs reduces the blastocyst formation rate and thus the live birth rate.

Sperm-specific RNAs may be epigenetic modifiers to affect the phenotype of the offspring. Microinjection of total RNAs from *Kit* ^tm1Alf/t^ mouse into fertilized eggs induced white tail in offspring [29]. Increasing evidences indicate that metabolic disorders in offspring are due to the father’s diet. Fasting of male mice before mating with female mice decreased serum glucose level in offspring [30] and the high-fat diet of male rats affected pancreatic islet biology in offspring [31]. The offspring from the normal zygotes injected with the total sperm RNAs or tRNAs from the male mice given a high-fat diet developed impaired glucose tolerance, showing higher blood glucose and serum insulin levels [32]. Moreover, differentially expressed sperm tsRNAs between control and high-fat diet males preferentially match to some gene promoter regions by sequence matches analysis, and biological pathway analysis showed that these matched genes are associated with diverse cellular and molecular processes, including apoptosis, autophagy, oxidative stress, glucose input (such as *Maea*, *Ccnc* and *Deptor* regulates the pancreatic β-cell function) [32]. In this study, RNases not only delete mRNAs, but also tRNAs. After the treatment with RNases, some sperm tRNAs, which affect genes involved in metabolism, may decrease, so the body weight of F1 mice from the treated sperm was lower than that from the control group.

In conclusion, the blastocyst formation rate and the live birth rate were decreased in embryos from ICSI using RNA-deficient sperm, and the body weight of F1 mice from the RNA-deficient sperm was lower than that from the control group. These results suggest that the sperm RNAs play critical roles in embryonic development.

## MATERIALS AND METHODS

All chemicals and media were purchased from Sigma Chemical Company (St. Louis, MO) except for those specifically mentioned.

### Animals

C57BL/6 mice were used to collect oocyte and sperm or to reproduce. Female mice mated with CD-1 vasectomized males were used as embryo recipients. Mice care and handling abided to the Animal Research Committee guidelines of the Institute of Zoology, Chinese Academy of Sciences.

### Preparation of spermatozoa

Cauda epididymal spermatozoa were dispersed into HTF medium. After incubation for 1h at 37°C in a CO2 incubator, the spermatozoa were washed once with HTF medium by centrifugation. Sperm pellets were resuspended in a medium consisting of 250mM NaCl, 100mM Tris-HCl, 10mM EGTA, 0.2% (w/v) lysolecithin, and 0.1% (w/v) pronase (PH7.8) and incubated at 37°C for 0.5h [18], then the sperm were treated with 5 mg/ml RNase A (TAKARA) and 400U/ml RNase H (TAKARA) at 37°C for 1.5h, followed by centrifugation. To remove the RNases, the pellets were washed six times in 500μl Hepes-CZB with each time for 2 mins, and then centrifugated 1min using 1000rpm. The spermatozoa were used for RNA isolation, or intracytoplasmic sperm injection (ICSI).

### RNA isolation and RNA-seq

The total sperm RNAs were extracted using TRIzol reagent (Invitrogen) following the manufacturer’s instructions including DNase treatment (Ambion). RNA was evaluated with a NanoDrop 6000 Labchip kit (Nanodrop) and an Agilent 2100 Bioanalyzer (Agilent Technologies, Santa Clara, CA, USA). Then the RNA was subjected to RNA sequencing following manufacturer’s recommendations (Novogene) using Illumina Hiseq 2500 instrument (Illumina, San Diego, CA, USA). Data collection and analysis was performed as previously described [33–35].

### Intracytoplasmic sperm injection (ICSI) and embryo culture

Intracytoplasmic sperm injection was carried out as described [36, 37]. 5μl sperm suspension was mixed with 10μl Hepes-CZB containing 10% (w/v) polyvinylpyrrolidone. A drop of this mixture was transferred into a plastic dish under paraffin oil previously placed on the objective table of an inverted microscope equipped with a Piezo-drill micromanipulation system. Two types of spermatozoa were injected into oocytes: 1) an intact sperm after washing with HTF medium, as control; 2) a sperm head after treatment with LL+P+RNases and washing with Hepes-CZB. The treated spermatozoa were used in 1h after treatment. For rescuing the reduction of sperm RNA, 1-2pl total sperm RNA (0.1 ng/μl) was injected into an oocyte before the treated sperm was injected. After ICSI, the oocytes were activated by Ca^2+^-free CZB containing 5 mM SrCl_2_ for 30 min, then cultured in KSOM^AA^ (Millipore) at 37°C in a CO_2_ incubator.

### Immunofluorescence and confocal microscopy

The process of immunoflurescence was performed as previously described [14, 38]. After fixed with 4% paraformaldehyde for 30 min and permeabilized for 30 min with 0.2% Triton X-100 in PBS and blocked in a blocking solution (1% BSA and 0.05% Tween-20 in PBS) for 2 h at room temperature, the embryos cultured 11h after ICSI or at 2-cell stage were then incubated with antibodies to H3K27me3 (Abcom), H3K4me3 (Epigentek), H3k14ac (Upstate) or H4k12ac (Upstate) overnight at 4°C. The samples were washed three times with washing buffer (PBS supplemented with 0.1% Tween 20 and 0.01% Triton X-100). Then the embryos were incubated with a fluorescein isothiocyanate (FITC)-conjugated goat anti-rabbit antibody (Zhong Shan Jin Qiao) for 1h at room temperature. Following three washes in washing buffer, DNA was stained with Hoechst 33342 (10 mg/ml), and all samples were mounted in antifade solution. The embryos were observed on a Zeiss LSM 780 microscope (Carl Zeiss). The embryos of the same developmental stage were photographed used the same instrument parameters. Nuclear intensities of integrated fluorescence were measured with the ImageJ 1.44p software as previously described [39]. All individual nuclei in embryos need outlined manually. After background subtraction, total fluorescence intensities of all individual nuclei in each embryo were obtained. The ratios of the fluorescence intensity of H3K27me3, H3K4me3, H3k14ac or H4k12ac to Hoechst 33342 DNA signal in the same embryo from ICSI using the sperm with or without LL+P+RNases-treated were compared. The images were managed using the Adobe Photoshop software (Adobe Systems, San Jose, CA) and no contrast or brightness were adjusted.

### Embryos transfer

Two-cell embryos were transferred into oviducts of CD-1 pseudopregnant females mated with vasectomized males at the previous night.

### Evaluation of reproductive capacity of offspring (F0) from the treated sperm

To evaluate the reproductive capacity, three female and three male offspring (F0) from the LL+P+RNases-treated sperm group or control group at the age of 8 week were mated with wide type C57BL/6 male or female mice, respectively. The numbers of pups and litters were recorded up to 6 months.

### Evaluation of body weight trajectories of offspring (F1) from the treated sperm

To examine the body weight trajectories, the F1 offspring in six litters from the LL+P+RNases-treated sperm group (F0) or control group (F0) were weighed every week for 8 weeks, respectively.

### Statistical analyses

Data were expressed as mean ± sem. Statistical differences between datasets were assessed by Student’s *t*-test or *chi*-squared test with the IBM SPSS Statistics 19.0 software. A value of *P*<0.05 was considered statistically significant.
